# Short-course antibiotic therapy: The next frontier in antimicrobial stewardship

**DOI:** 10.14745/ccdr.v48i1112a01

**Published:** 2022-11-03

**Authors:** Donald Sheppard

**Affiliations:** 1 Antimicrobial Resistance Task Force, Public Health Agency of Canada, Ottawa, ON; 2 Department of Microbiology and Immunology, Faculty of Medicine, McGill University, Montréal, QC; 3 Infectious Disease and Immunity in Global Health, Research Institute of McGill University Health Center, Montréal, QC; 4 McGill Interdisciplinary Initiative in Infection and Immunity, Montréal, QC

**Keywords:** antibiotic therapy, duration, antimicrobial, stewardship, short-course antibiotherapy, antimicrobial resistance

## Abstract

Ensuring appropriate use of antibiotics is critical to preserving their effectiveness through limiting the development and spread of antimicrobial resistance. Evidence is accumulating that shorter courses of antibiotics are as effective as traditional longer regimens for many common infections and can reduce the risk of adverse events. Despite the availability of evidence and guidelines supporting short-course antibiotic therapy for these conditions, prolonged use of antibiotics remains common. This article will review the origins and evolution of our approach regarding antimicrobial prescription duration, the evidence for the use of short-course therapy for selected infections, barriers to the uptake of this practice and potential approaches that can be taken to reduce inappropriately long antibiotic use.

## Introduction

Antibiotics have transformed modern medicine, but their continued viability is threatened by rising rates of antimicrobial resistance (AMR). Limiting inappropriate use of antibiotics is an important approach to reducing the antibiotic pressure that can accelerate the evolution and spread of AMR. Evidence is emerging that for many common infections, short-course antibiotic therapy (1–7 days) can be equally as effective as traditional, longer courses of treatment ((1,2)). Expanded use of short-course antibiotic therapy has the potential to reduce healthcare costs, reduce risks of adverse drug events and help curb AMR. This editorial will review the history of the development of antibiotic treatment duration, highlight key evidence for appropriate reductions in antibiotic therapy duration, and outline future directions in antimicrobial stewardship and knowledge generation that could support the reduction of unnecessary prolonged antibiotic therapy.

### Origins of the current approach to antibiotic therapy duration

The modern antibiotic era began with the introduction of penicillin in 1940, and with it, the dilemma of determining the appropriate duration of therapy for infectious diseases. Albert Alexander, a British constable, was the first human to receive penicillin therapy for extensive streptococcal and staphylococcal facial abscesses following an injury sustained in a bombing raid ((3)). Constable Alexander received five days of penicillin therapy with an excellent short-term response; however, despite attempt to re-purify penicillin from his urine, the supply of purified penicillin was exhausted and therapy was discontinued as a result. Sadly, within several weeks, his infection returned and he eventually succumbed to his disease. While this first use of penicillin demonstrated the power of antibiotics to treat bacterial infections, it also presaged the current challenge of determining antibiotic therapy duration, highlighting both the question of “how much is enough”, and the legitimate fear that inadequate therapy may result in relapse or poorer outcomes.

Despite this somewhat disheartening first experience with short-course antibiotic therapy, early prescribers of penicillin reported that 1.5–4 days of treatment was sufficient to cure the majority of patients with diseases like pneumococcal pneumonia ((1)). Indeed, one of the earliest trials of penicillin therapy for pneumococcal pneumonia demonstrated that when therapy was discontinued 2–3 days after clinical improvement and resolution of fever, only 3 of 54 patients relapsed after initial therapy ((4)). One of these cases occurred in a patient receiving only 24 hours of therapy, and in the other two cases the strain at relapse was found to be a different serotype than the original infecting isolate, suggesting a reinfection rather than a relapse ((4)). Collectively these observations suggest that longer courses of 1–2 weeks of penicillin for pneumonia are unnecessary for the majority of patients.

Many factors have likely influenced the shift from this original approach of using antibiotics for short courses, tailored to patient responses, to the modern, longer, 1–2 weeks fixed duration of therapy ((1)). The rise in outpatient care and the shift away from intensive patient follow-up makes daily assessment of response to therapy less practical and favours the use of fixed duration prescriptions. Experience with infections that require longer term antibiotic therapy like tuberculosis and endocarditis may have influenced attitudes towards the duration of therapy required for all infectious diseases. Public perceptions of risk and the current medico-legal climate have also combined to create a culture of caution in modern medicine. As antibiotics are largely well tolerated and safe, there is always the temptation to extend prescription duration to reduce perceived risks of relapse. Finally, and perhaps most importantly, a perceived lack of rigorous evidence supporting shorter course antibiotic therapy limits prescriber confidence in breaking with traditional longer, fixed duration therapies ((5)).

### The case for shortening antimicrobial duration of therapy in selected infections

Although antibiotics are commonly viewed as “safe” medications, the potential advantages of prolonging antibiotic therapy duration must be weighed against the costs and potential for harm. From an economic perspective, it has been estimated that the cost of antimicrobial prescribing in Canada exceeds $750 million per year ((6)). It is evident that reducing the duration of antibiotic therapy has significant potential to reduce these costs. Prolonged antibiotic therapy can also increase the chance of medication for adverse events or drug-drug interactions and has been linked to increased risk of *Clostridioides difficile* infection ((7)). One study reported that in patients with pneumonia receiving continued antibiotic therapy after discharge, each excess day of treatment was associated with a 5% increase in the odds of self-reported antibiotic-associated adverse events ((8)). Beyond these direct costs and risks, longer courses of antibiotic therapy have been linked to an increased burden of resistance ((9)). In a study of antibiotic therapy for ventilator-associated pneumonia, recurrent infections with multidrug-resistant organisms were more commonly observed in patients receiving antibiotic prescriptions of 18 days as compared with those receiving only eight days ((10)). These data may suggest a need to revisit the broad use of public health messaging that encourages patients to complete their course of antibiotic therapy even after they feel better. For many conditions, this practice may be unnecessary and actually favour the emergence of resistance ((1)).

Clinical trials comparing short- and long-course therapy are accumulating, and a common theme is emerging supporting equivalent or better outcomes with short-course therapy. Multiple trials have demonstrated that short-course therapy (1–3 days) is highly effective for the treatment of uncomplicated urinary tract infection ((11,12)), and that pyelonephritis and urosepsis in adults can be treated with seven days of an appropriate agent ((13–15)). Similarly, studies in both community and hospital-acquired pneumonia comparing the efficacy of short-course (5–7 days) therapy have found equivalent efficacy, and reduced rates of adverse events when compared with longer courses of treatment ((8,16,17)). The efficacy of short-course therapy extends to severe infections as well. Three randomized controlled clinical trials have demonstrated the safety and efficacy of seven days of antibiotic therapy for bacteremia with gram-negative bacilli ((18–21)). A single large study has even challenged the dogma that antibiotic therapy for the treatment of febrile neutropenia must be continued until neutrophil recovery ((22)). This trial reported that antibiotic therapy for febrile neutropenia could be safely discontinued in patients with resolution of fever and clinical recovery, irrespective of their neutrophil counts ((22)). In recognition of the mounting evidence for short-course therapy, the Association of Medical Microbiology and Infection Disease Canada has recently published a practice point summary of duration of antibiotic therapy for common infections highlighting recent evidence for reduced duration of antibiotic treatment in select infectious diseases syndromes ((23)).

While the majority of trials on the duration of antibiotic therapy have supported the use of short-course therapy, there are some notable exceptions. A single trial reported that a six-week course of antibiotic therapy had inferior outcomes than a 12 weeks for prosthetic joint infection ((24)), although a second trial found that eight weeks was equally effective as longer course therapy for early prosthetic joint infection ((25)). Several meta-analyses of treatment trials of streptococcal pharyngitis found that bacterial eradication rates were higher with 10-day courses of penicillin; these differences were less marked with non-penicillin antibiotic treatments ((26–28)). Finally, although trials of treatment for otitis media in children found that 5–7 days of antibiotic therapy was effective ((29)), a single trial in children under the age of two years reported that five days of therapy was less effective than 10 days ((30)). A detailed list of these and other studies of antibiotic treatment duration has been curated by Dr. Brad Spellberg, University of Southern California.

### Awareness of evidence in support of short-course antibiotic therapy: a knowledge mobilization opportunity

Despite the availability of new recommendations and position pieces from professional associations, adherence to best practices in reducing inappropriately long antibiotic prescriptions remains suboptimal. International studies have reported high levels of unnecessarily prolonged antibiotic therapy in both primary care and hospital settings. A review of primary care data in England from 2013 to 2015 recorded an estimated 1.3 million days of excess antibiotic prescriptions ((31)). Similarly, a study of pneumonia treatment in the United States revealed that as many as two out of three patients received excess antibiotic therapy ((8)). Canadian data on the appropriateness of the duration of antibiotic prescriptions are relatively sparse. A recent report on a stewardship intervention in primary care reported that 29.3% of prescriptions for community-acquired infections were inappropriately long (defined as more than seven days) ((32)). This report likely underestimates the degree of inappropriately prolonged prescription as it included cystitis, for which three days of antibiotic therapy is the standard of care. Similar findings have been reported in long-term care centres; a province-wide review of antimicrobial use in long-term care found that 44.9% of prescriptions exceeded seven days’ duration ((33)). A limited number of studies have also identified high levels of prolonged antibiotic therapy in the Canadian hospital setting. An early study of treatment for hospital-acquired pneumonia found that only 30% of patients were treated with an appropriate duration of antibiotics ((34)). A second retrospective survey of the treatment of ventilator-associated pneumonia in a large Canadian urban health region found that more than 50% of patients received inappropriately prolonged antibiotic therapy ((35)). Collectively, these data suggest there is significant room for improvement in ensuring appropriate duration of antibiotic therapy in both the Canadian community and hospital sectors.

Behavioural science studies are beginning to shed light on the drivers underlying the continued use of prolonged antibiotic treatments by prescribers. International trials have suggested that prescriber preference and habit, rather than patient characteristics are the primary determinant of duration of antibiotic prescription trials ((36)), an observation that was replicated in the Canadian long-term care setting ((33)). Building on these findings, a recent behaviour change analysis in Canadian long-term care institutions highlighted a number of barriers to improving uptake of short-term antibiotic therapy, including a perceived lack of evidence, the often incorrect belief that short-course therapy could increase rates of antimicrobial resistance, as well as the previously documented strong effects of prior habits and belief in guiding prescription behaviours ((5)).

There are multiple approaches that could be taken to improve the uptake of short-course antibiotics in Canada. Increasing the awareness of new guidelines for short-course antibiotic therapies should be a goal of stewardship programs and targeted awareness campaigns and should be incorporated into professional education and maintenance of competence. There is evidence that these types of stewardship interventions can improve appropriate antibiotic prescription duration. Use of a multifaceted program of clinician education, clinical decision aids, patient information and audit and feedback in the Canadian outpatient setting resulted in significantly lower rates of inappropriately long-prescription duration as compared with clinics that did not receive the intervention ((32)). In parallel, the inclusion of measures of appropriateness of the duration of antibiotic use in antimicrobial use surveillance and epidemiologic studies will be critical in identifying populations and settings where prolonged antibiotic use is high, as well as monitoring the effectiveness of interventions and awareness campaigns designed to reduce this overuse. Traditionally, most surveillance programs and epidemiologic studies of antimicrobial use have focused on quantitative measures of total antibiotic use and quality measures that are driven by matching diagnoses to prescriptions, but often do not capture the duration of therapy by indication. Looking forward, although evidence is slowly emerging that the mantra of “short is better” is often correct, it is by no means a universal truth. Further studies are required to validate some of the seminal studies referenced here, and to explore the appropriate duration of therapy for other infectious diseases where prolonged antibiotic therapy has been linked to the emergence of resistance, such as sternal surgical infections following cardiac surgery ((37)). As evidence emerges, it may be possible to develop a better scientific framework to guide our understanding of what factors determine the need for prolonged antibiotic therapy to allow better identification of clinical predictors that can guide prescription duration in specific patient populations. Finally, expanding behavioural science research to better understand the barriers and enablers to implementing short-course antibiotic therapy has the potential to guide the development of novel approaches to improve rates of appropriate antibiotic therapy duration. The potential of behavioural science to guide effective stewardship initiatives has been clearly demonstrated in the United Kingdom at the national scale. In 2014, social norm feedback was provided to high prescribers of antibiotics in the form of a letter from England’s Chief Medical Officer, accompanied by a leaflet on appropriate antibiotic use ((38)). This single intervention resulted in a sustained 3.3% reduction in antibiotic prescriptions, approaching the level of the five-year United Kingdom target of a 4% reduction of antibiotic use in primary care ((38)).

## Conclusion

Ensuring antibiotics are used for an appropriate duration has the potential to reduce cost, improve patient outcomes and reduce antimicrobial resistance. There are multiple opportunities to advance the use of short-course therapy in clinical infectious diseases in Canada, including 1) improving awareness and education of existing duration of therapy guidelines, 2) implementing effective surveillance for appropriateness of antimicrobial prescription duration and 3) conducting studies to identify both the optimal length of therapy across a wide range of infectious diseases syndromes and the behavioural factors underlying prescriber practises in order to guide interventions aimed at reducing inappropriately long antibiotic prescriptions.
